# Videos in short-video sharing platforms as sources of information on heatstroke: a cross-sectional content analysis study

**DOI:** 10.3389/fpubh.2026.1714828

**Published:** 2026-04-09

**Authors:** Zhiqiang Liao, Zhimin Qi, Xingzi Shangguan, Mengting Huang, Chong Shan, Zhidong Zhou

**Affiliations:** 1 Department of Anesthesiology, The Second Affiliated Hospital, Jiangxi Medical College, Nanchang University, Nanchang, Jiangxi, China; 2 Jiangxi Province Key Laboratory of Anesthesiology, Nanchang, Jiangxi, China; 3 School of Stomatology, Jiangxi Medical College, Nanchang University, Nanchang, China; 4 Jiangxi Province Key Laboratory of Oral Diseases & Jiangxi Province Clinical Research Center for Oral Diseases, Nanchang, China

**Keywords:** health information, heatstroke, quality assessment, reliability, short videos

## Abstract

**Background:**

Over the past few years, short videos have shown considerable promise as a medium for disseminating health-related information. Health-related content about heatstroke is extensively circulated across short video platforms. Nonetheless, the quality, credibility, practical value, and accuracy of the professional knowledge conveyed in these short videos have not been systematically assessed.

**Objective:**

This study aims to analyze the content and quality of videos related to heatstroke on short video sharing platforms.

**Methods:**

As of September 1, 2025, the term “heatstroke” was used as a keyword to search on TikTok, BiliBili, and Kwai short video platforms, and the top 300 videos from each platform were included and recorded. Two qualified researchers independently assessed the content and quality of the selected videos utilizing the Journal of the American Medical Association (JAMA) scoring system, the Global Quality Scale (GQS), the modified DISCERN instrument, and the Patient Education Materials Assessment Tool (PEMAT). SPSS version 26.0 and decision chain analysis were used to generate descriptive statistics, compare differences between groups, and assess relationships among variables via Spearman correlation analysis.

**Results:**

This study analyzed 632 heatstroke-related videos on BiliBili, TikTok, and Kwai. The quality of videos varied considerably across platforms. These videos had a mean JAMA score of 1.50 (SD: 0.72), a mean GQS score of 3.14 (SD: 0.83), a mean modified DISCERN score of 2.35 (SD: 0.74), a mean PEMAT-understandability score of 0.58 (SD: 0.11), and a mean PEMAT-actionability score of 0.50 (SD: 0.32). Overall, the general quality and reliability of videos on TikTok and BiliBili were superior to those on Kwai. Most videos were uploaded by news agencies and physicians (accounting for 37.5% and 35.28%, respectively), with the content primarily focusing on symptoms (32.75%) and treatment (23.73%). Across platforms, video duration was positively correlated with video quality.

**Conclusions:**

The findings reveal that heat stroke-related short videos across BiliBili, TikTok, and Kwai are generally of low quality and vary markedly among platforms, which may misguide public health practices. The results suggest the need to strengthen the development of authoritative science popularization content, optimize health communication strategies, and introduce platform quality assessment and recommendation mechanisms to enhance the public's disease prevention capabilities.

## Introduction

Heatstroke, a life-threatening systemic illness induced by heat stress, is primarily manifested by profound hyperthermia (typical core body temperature ≥ 40.5 °C) and is often accompanied by systemic inflammatory response syndrome (SIRS) as well as multiple organ dysfunction syndrome (MODS), among other complications (1). Etiologically, heatstroke is categorized into classic heatstroke and exertional heatstroke, with the former occurring mainly in populations with reduced immune function, and the latter frequently affecting athletes and outdoor workers exposed to high-temperature conditions (2). In the context of global temperature rise, the yearly mortality rate from heat-related causes among individuals over the age of 65 has risen by about 68% in the last two decades (3). Global statistics show that in 2023, heat-related illnesses (HRI) increased significantly worldwide. In some parts of the United States alone, HRI accounted for about 120,000 medical visits, with an average hospitalization cost of $17,372, representing a considerable socioeconomic and healthcare burden (4). Under intensive care conditions, mortality rates still reach 26.5% for exertional heatstroke and 63.2% for classic heatstroke (1). Given that heat stroke is a preventable disease, the three-level prevention strategy (reducing risk factors, early detection and intervention, treatment and rehabilitation management) is considered key to mitigating its harm (3).

As the Internet and social media continue to expand rapidly, short video platforms have emerged as a vital source of health information for the public, owing to the ease of creating and consuming such content. According to statistics, more than 3.8 billion people globally use social media, with an average daily usage time of 2.4 h per user (5). By June 2025, China had an internet penetration rate of 79.7%, a total of 1.123 billion netizens, and 1.068 billion of them were short video platform users (6). TikTok, BiliBili, and Kwai rank as the top three short video platforms in China by user scale, offering representative coverage across diverse age demographics and content genres. Overall, the average monthly time spent per person on short videos is 62.9 h; TikTok and Kwai report monthly active users of 978 million and 587 million respectively (7). However, at present, short video platforms have not yet implemented strict regulation of health-related videos, and inaccurate or even false health information videos still account for a certain proportion of users' viewing content, thereby affecting individual health decisions. Therefore, it is necessary to assess the content, accuracy, and quality of health information short videos. Existing studies have explored the quality of online health information on social media and short-video platforms, such as assessments of accuracy and credibility for specific diseases like bipolar disorder (8) and esophageal cancer (9), as well as quality analyses of aerobic exercise training videos for patients with diabetes (10), thereby providing practical references for video quality assessment across diverse health topics (e.g., disease prevention, rehabilitation guidance). However, investigations into content quality specifically for heat stroke—a preventable disease with high mortality—are still needed, especially regarding systematic analyses on China's dominant platforms (TikTok, BiliBili, and Kwai).

Accordingly, this research seeks to assess the quality and credibility of heat stroke-related videos on leading short video platforms (TikTok, BiliBili, and Kwai), employing internationally standardized metrics for quantitative analysis, investigating disparities among platforms and associated determinants, so as to offer specific guidance for health communication initiatives and platform content regulation, while facilitating the use of short video platforms as efficient instruments for improving public knowledge and preventive capacity against heat stroke.

## Methods

### Ethical consideration

All data in this study were sourced from public posts on TikTok, BiliBili, and Kwai, containing no personal privacy details and involving no use of human samples, laboratory animals, or clinical data. Consequently, no ethical approval is necessary.

### Search strategy and data collection

**Figure 1:**
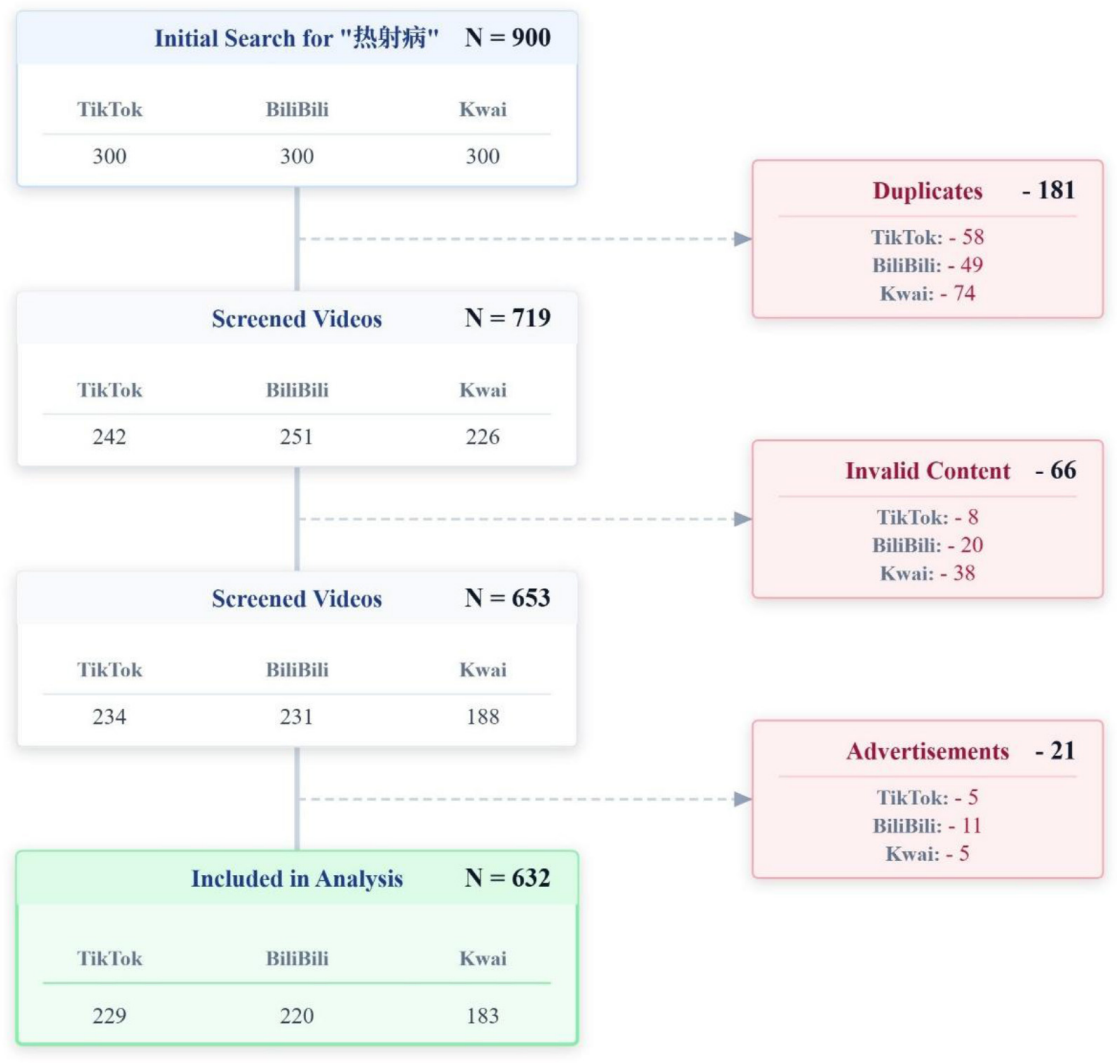
Search strategy and video screening procedure.

Searches were performed independently on TikTok, BiliBili, and Kwai, ending on September 1, 2025. Currently, platform algorithms can map a keyword (heat stroke) to its relevant knowledge system and perform comprehensive ranking based on multiple dimensions such as relevance, authority, and timeliness, thereby delivering thorough search results (11). To reduce the impact of algorithmic biases toward personal interests, new accounts were registered prior to searching the term “热射病” (“heatstroke”) on all three short video platforms. Considering that the three platforms primarily employ comprehensive sorting for displaying search results by default, and prior research indicates that most internet users prefer to view content ranked higher in search listings, we therefore collected the top 300 videos by comprehensive ranking on each platform (12). Inclusion criteria were: (1) Chinese-language videos; (2) heatstroke-related videos. Exclusion criteria were: (1) irrelevant content; (2) duplicate videos; (3) advertisements. As illustrated in Figure 1, the short video collection process began with 300 videos from each of the three major platforms. Following the removal of duplicates (*N* = 181), unrelated content (*N* = 66), and advertisements (*N* = 21), 632 heat stroke-related videos were retained: 229 from TikTok, 220 from BiliBili, and 183 from Kwai. For each collected video, detailed information was recorded, including title, number of likes, comments, favorites, shares, days since upload, video length, source, uploader's follower count, presentation style, and content; detailed classification is provided in Supplementary Table S1.

### Assessment of content and quality of videos

The JAMA benchmark criteria, established by the Journal of the American Medical Association, include four key components: authorship, attribution, disclosure, and timeliness, which serve to evaluate the trustworthiness of health-related online information (13). The Global Quality Scale (GQS) employs five assessment items to comprehensively determine a video's overall quality from the perspectives of accuracy, authority, and completeness, scoring from 1 (poor) to 5 (excellent in flow and quality) (14). The modified DISCERN tool assesses aspects such as relevance, source traceability, and scientific evidence of health information through five specific questions (15). Beyond measuring short video quality and reliability, we applied the Patient Education Materials Assessment Tool (PEMAT) to analyze their clarity and actionability, enabling a more holistic evaluation (16). Full scoring guidelines for these assessment tools can be found in Supplementary Tables S2–S5.

To ensure the accuracy and reliability of the scoring process, we conducted standardized training for the reviewers prior to evaluation and calibrated them using trial videos. All videos were gathered and downloaded by one person (CS). After randomizing the video order, two senior physicians (ZQ-L and ZM-Q) independently scored the videos, with the final scores determined by an adjudicator (ZD-Z). Additionally, reliability between and within raters was assessed using the intraclass correlation coefficient (ICC), where values below 0.50 indicate poor reliability, between 0.50 and 0.75 indicate moderate reliability, between 0.75 and 0.90 indicate good reliability, and above 0.90 indicate excellent reliability (17). Inter-rater agreement was good: JAMA score (ICC = 0.91, 95% CI 0.89–0.94), GQS score (ICC = 0.83, 95% CI 0.80–0.84), modified DISCERN score (ICC = 0.76, 95% CI 0.73–0.79), understandability (ICC = 0.81, 95% CI 0.78–0.83), and actionability (ICC = 0.87, 95% CI 0.85–0.89). Intra-rater reliability also remained high (JAMA score = 0.98, GQS score = 0.86, modified DISCERN score = 0.79, understandability = 0.80, actionability = 0.87).

### Statistical analysis

Data analysis was conducted using SPSS 22.0 (IBM Corporation, Armonk, NY, USA). Categorical data were presented as counts and percentages. Distribution features of continuous variables were initially examined via the Shapiro-Wilk test along with Q-Q plots and histograms: those conforming to a normal distribution were reported as mean ± standard deviation (Mean ± SD), whereas those deviating from normality were described using median and range [Median (Range)]. For comparisons between two groups, either the Mann–Whitney U test (non-normal distribution) or Student's *t*-test (normal distribution) was applied; for three-group comparisons, the Kruskal–Wallis test or one-way ANOVA was used. For multiple group comparisons, the Bonferroni method was used to correct *p*-values for multiple comparisons in order to control the Type I error rate. Relationships between quantitative variables were assessed using Spearman's rank correlation. Statistical significance was set at *P* < 0.05.

## Results

### The general characteristics of videos

Table 1 indicates that Kwai's videos outperformed those on TikTok and BiliBili in views, likes, favorites, shares, and comments (*P* < 0.001). The predominant video source varied across platforms—news agencies on Kwai (60.11%), certified doctors on TikTok (62.73%), and independent users on BiliBili (42.36%). On TikTok, expert monologues dominated (60.00%), while BiliBili and Kwai tended to use visual imagery (47.16% and 68.13%). TikTok and BiliBili outscored Kwai on JAMA, GQS, modified DISCERN, understandability and actionability.

**Table 1:** The general characteristics and scoring situation of videos related to heat stroke.

Variables	Total	TikTok	BiliBili	Kwai	*p*-value
	(*N* = 632)	(*N* = 220)	(*N* = 229)	(*N* = 183)	
View counts, median (range)	41,409.50 (4.00–46,021,299.00)	41,409.50 (502.00–46,021,299.00)	2,221.00 (4.00–2,013,000.00)	276,081.00 (7,409.00–17,202,466.00)	< 0.001
Likes, median (range)	510.50 (0.00–628,847.00)	351.50 (3.00–628,847.00)	49.00 (0.00–67,000.00)	2,369.00 (64.00–172,549.00)	< 0.001
Collections, median (range)	79.00 (0.00–199,900.00)	60.50 (0.00–199,900.00)	28.00 (0.00–15,000.00)	224.00 (1.00–20,000.00)	< 0.001
Shares, median (range)	139.00 (0.00–1,176,487.00)	145.00 (0.00–1,176,487.00)	14.00 (0.00–9,713.00)	896.00 (15.00–272,000.00)	< 0.001
Days since upload, median (range)	60.00 (4.00–1,530.00)	59.00 (26.00–1,149.00)	104.00 (4.00–1,530.00)	/	0.001
Duration, median (range)	77.00 (0.00–9,375.00)	79.50 (7.00–329.00)	129.00 (0.00–9,375.00)	16.00 (4.00–284.00)	< 0.001
Comments, median (range)	26.00 (0.00–58,968.00)	19.00 (0.00–58,968.00)	4.00 (0.00–7,349.00)	142.00 (1.00–27,161.00)	< 0.001
15.6-7.4,-14498pt Fans, median (range)	245,394.50 (0.00–174,910,041.00)	572,748.50 (10.00–174,910,041.00)	14,000.00 (0.00–8,127,000.00)	1,582,000. (300.0–16,160,000.0)	< 0.001
Video source, *n* (*p*%)					< 0.001
Physicians	223.00 (35.28%)	138.00 (62.73%)	65.00 (28.38%)	20.00 (10.93%)	
Independent users	164.00 (25.95%)	14.00 (6.36%)	97.00 (42.36%)	53.00 (28.96%)	
News agencies	237.00 (37.50%)	67.00 (30.45%)	60.00 (26.20%)	110.00 (60.11%)	
15.6-7.4,-14498pt Others	8.00 (1.27%)	1.00 (0.45%)	7.00 (3.06%)	0.00 (0.00%)	
Different medical specialties, *n* (*p*%)					< 0.001
Western medicine practitioner	196.00 (31.01%)	122.00 (55.45%)	56.00 (24.45%)	18.00 (9.84%)	
15.6-7.4,-14498pt Traditional chinese medicine practitioners	16.00 (2.53%)	7.00 (3.18%)	8.00 (3.49%)	1.00 (0.55%)	
Video content, *n* (*p*%)					< 0.001
Disease knowledge	451.00 (71.36%)	192.00 (87.27%)	184.00 (80.35%)	75.00 (40.98%)	
Personal experience	179.00 (28.32%)	27.00 (12.27%)	44.00 (19.21%)	108.00 (59.02%)	
15.6-7.4,-14498pt Others	2.00 (0.32%)	1.00 (0.45%)	1.00 (0.44%)	0.00 (0.00%)	
Different disease knowledge, *n* (*p*%)					< 0.001
Symptom	207.00 (32.75%)	84.00 (38.18%)	76.00 (33.19%)	47.00 (25.68%)	
Treatment	150.00 (23.73%)	84.00 (38.18%)	53.00 (23.14%)	13.00 (7.10%)	
Prevention	67.00 (10.60%)	26.00 (11.82%)	22.00 (9.61%)	19.00 (10.38%)	
Pathogenesis	87.00 (13.77%)	19.00 (8.64%)	55.00 (24.02%)	13.00 (7.10%)	
Definition	29.00 (4.59%)	2.00 (0.91%)	12.00 (5.24%)	15.00 (8.20%)	
15.6-7.4,-14498pt Others	19.00 (3.01%)	5.00 (2.27%)	11.00 (4.80%)	3.00 (1.64%)	
Video presentation form, *n* (*p*%)					< 0.001
Expert monologue	210.00 (33.23%)	132.00 (60.00%)	51.00 (22.27%)	27.00 (14.75%)	
Visual pictures and literature	279.00 (44.15%)	46.00 (20.91%)	108.00 (47.16%)	125.00 (68.31%)	
Vlogs of patients	1.00 (0.16%)	0.00 (0.00%)	1.00 (0.44%)	0.00 (0.00%)	
Dialogue	23.00 (3.64%)	9.00 (4.09%)	8.00 (3.49%)	6.00 (3.28%)	
Animation	36.00 (5.70%)	4.00 (1.82%)	28.00 (12.23%)	4.00 (2.19%)	
Others	83.00 (13.13%)	29.00 (13.18%)	33.00 (14.41%)	21.00 (11.48%)	
JAMA score, mean (SD)	1.50 (0.72)	1.76 (0.45)	1.72 (0.74)	0.91 (0.60)	< 0.001
GQS score, mean (SD)	3.14 (0.83)	3.45 (0.71)	3.34 (0.63)	2.54 (0.88)	< 0.001
Modified DISCERN score, mean (SD)	2.35 (0.74)	2.60 (0.50)	2.56 (0.76)	1.78 (0.63)	< 0.001
Understandability, mean (SD)	0.58 (0.11)	0.62 (0.08)	0.60 (0.10)	0.51 (0.13)	< 0.001
Actionability, mean (SD)	0.50 (0.32)	0.62 (0.28)	0.60 (0.29)	0.23 (0.25)	< 0.001

**Figure 2:**
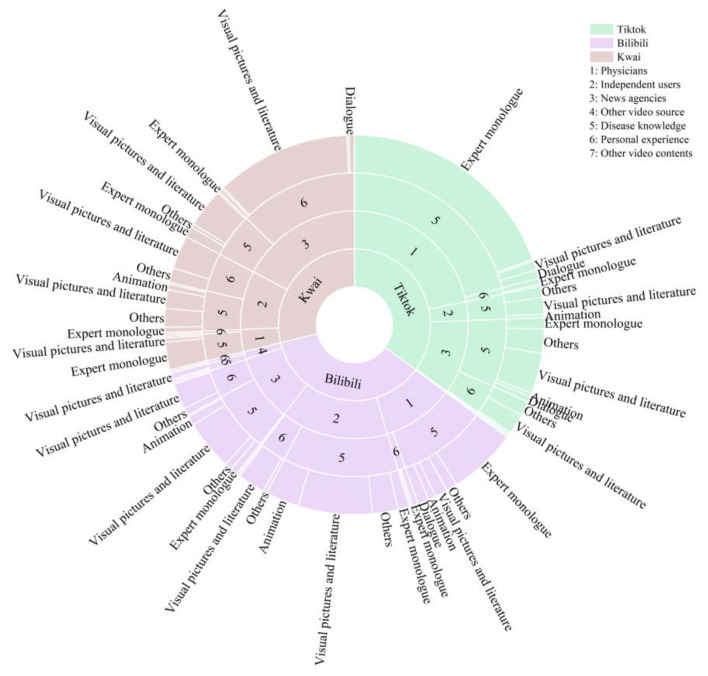
Comparative distribution of video sources, content types, and presentation styles among the three leading short video platforms.

Additionally, a multi-ring chart analysis (Figure 2) highlighted how TikTok, BiliBili, and Kwai differ in the distribution of video sources, content types, and presentation styles. On TikTok, the dominant share of content comes from “physicians” as the main source, using “expert monologue” formats to present “disease knowledge.” On Kwai, “news agencies” act as the primary information suppliers, favoring “visual graphic” formats that center on “personal experience” content creation. On BiliBili, “independent users” lead content production, with “visual imagery” used predominantly to deliver “disease knowledge,” forming the core of traffic and creative efforts.

### Quality and popularity of videos from different sources

**Figure 3:**
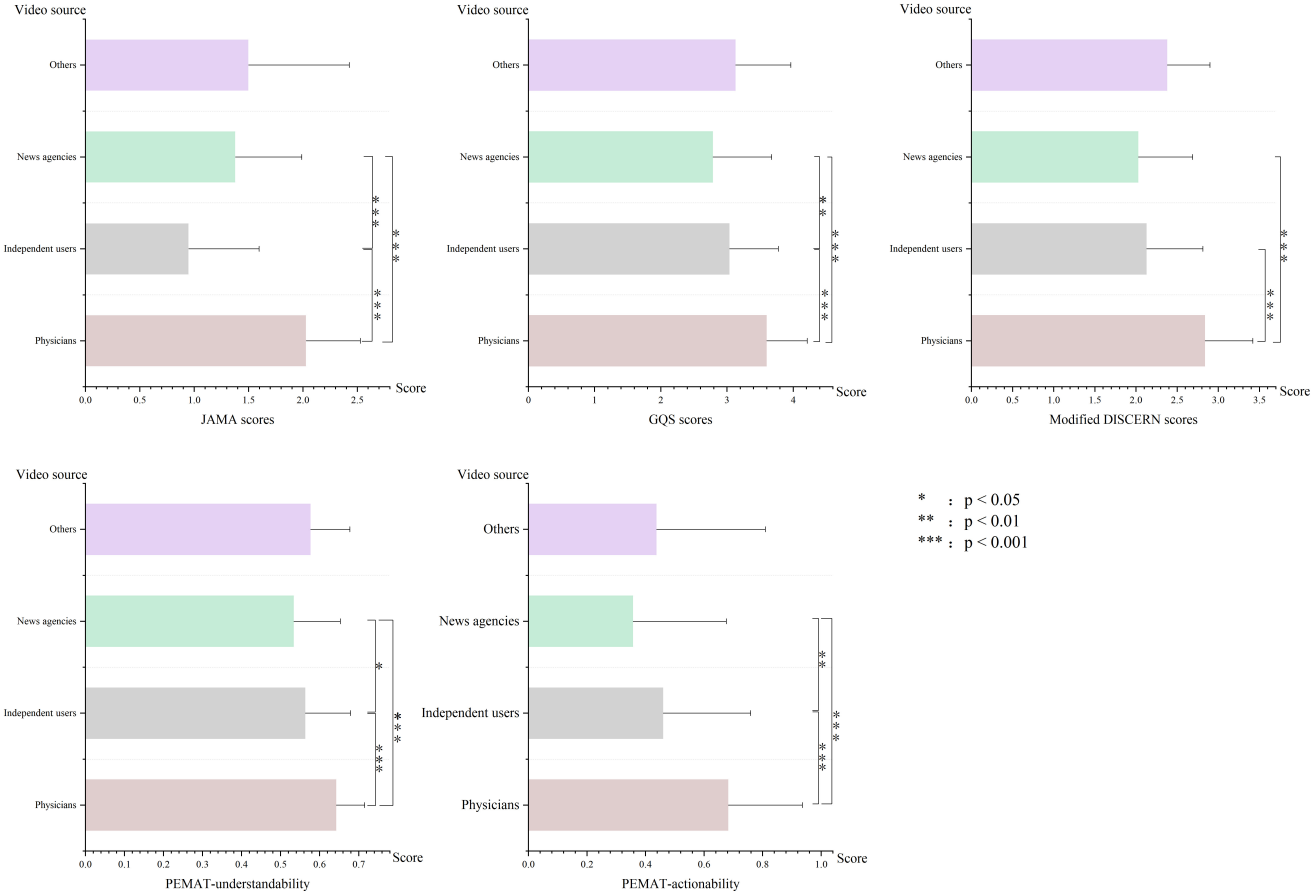
Score comparison across different video sources. ^*^p < 0.05, ^**^p < 0.01, ^***^p < 0.001.

Figure 3 presents a systematic assessment of the content quality (JAMA, GQS, modified DISCERN) and patient guidance value (PEMAT-actionability, PEMAT-understandability) of videos from four source types: news agencies, independent users, physicians, and others. The data reveal that videos produced by physicians scored significantly higher than those from independent users and news agencies on all metrics (all *P* < 0.001). News agency videos also achieved higher JAMA scores than those from independent creators (all *P* < 0.001), suggesting stronger scientific accuracy and practical applicability. News agencies placed second, surpassing independent users and miscellaneous sources in overall quality, though remaining slightly behind physicians—likely due to their capacity for professional resource integration. Videos from independent users and other miscellaneous sources showed comparatively poor quality, with lower median scores and greater variability (boxplots displaying broader IQRs and long tails), indicating a general deficiency in accuracy, structural completeness, and practical usefulness among non-professional or mixed-source content.

### Quality of videos on different platforms

**Figure 4:**
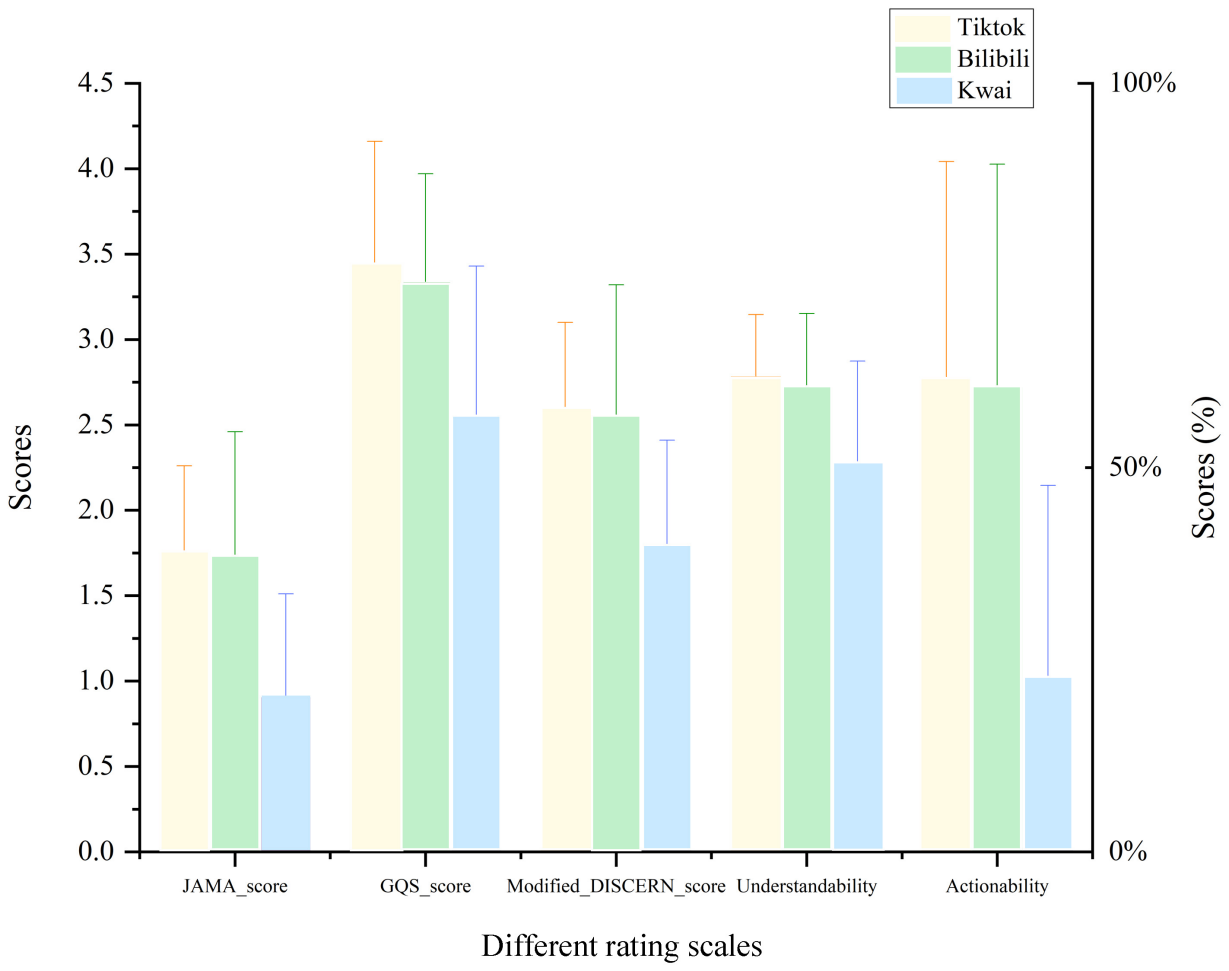
Performance comparison of the three platforms across various evaluation dimensions.

In evaluating the quality of heatstroke-related short videos, TikTok, BiliBili, and Kwai exhibited significant differences (*P* < 0.001) in five key metrics: JAMA, GQS, modified DISCERN, understandability, and actionability. TikTok ranked highest across all evaluation criteria, with BiliBili a close second; the two showed no significant differences in most areas (*P* > 0.05), yet both significantly outperformed Kwai (*P* < 0.001). Kwai's video content was markedly inferior to TikTok and BiliBili in information authority (JAMA), overall quality (GQS), evidence support (DISCERN), clarity, and action-oriented guidance (Figure 4 and Table 2).

**Table 2:** Performance comparison of TikTok, BiliBili, and Kwai in various evaluation metrics.

Categories	JAMA	GQS	Modified DISCERN	Understandability	Actionability
TikTok	1.76 (0.50)	3.45 (0.71)	2.60 (0.50)	61.92% (8.13%)	61.82% (28.00%)
BiliBili	1.72 (0.74)	3.34 (0.63)	2.56 (0.76)	60.06% (10.03%)	60.48% (28.57%)
Kwai	0.91 (0.60)	2.54 (0.89)	1.78 (0.63)	50.86% (13.05%)	22.68% (24.68%)
K-W: P	0.000	0.000	0.000	0.000	0.000
MW W: B vs. T	0.060	0.089	0.174	0.081	0.618
B vs. K	0.000	0.000	0.000	< 0.001	0.000
T vs. K	0.000	0.000	0.000	0.000	0.000

### Analysis of correlations between variables across different short video platforms

#### TikTok: the significant counter-effect between quality and flow

**Figure 5:**
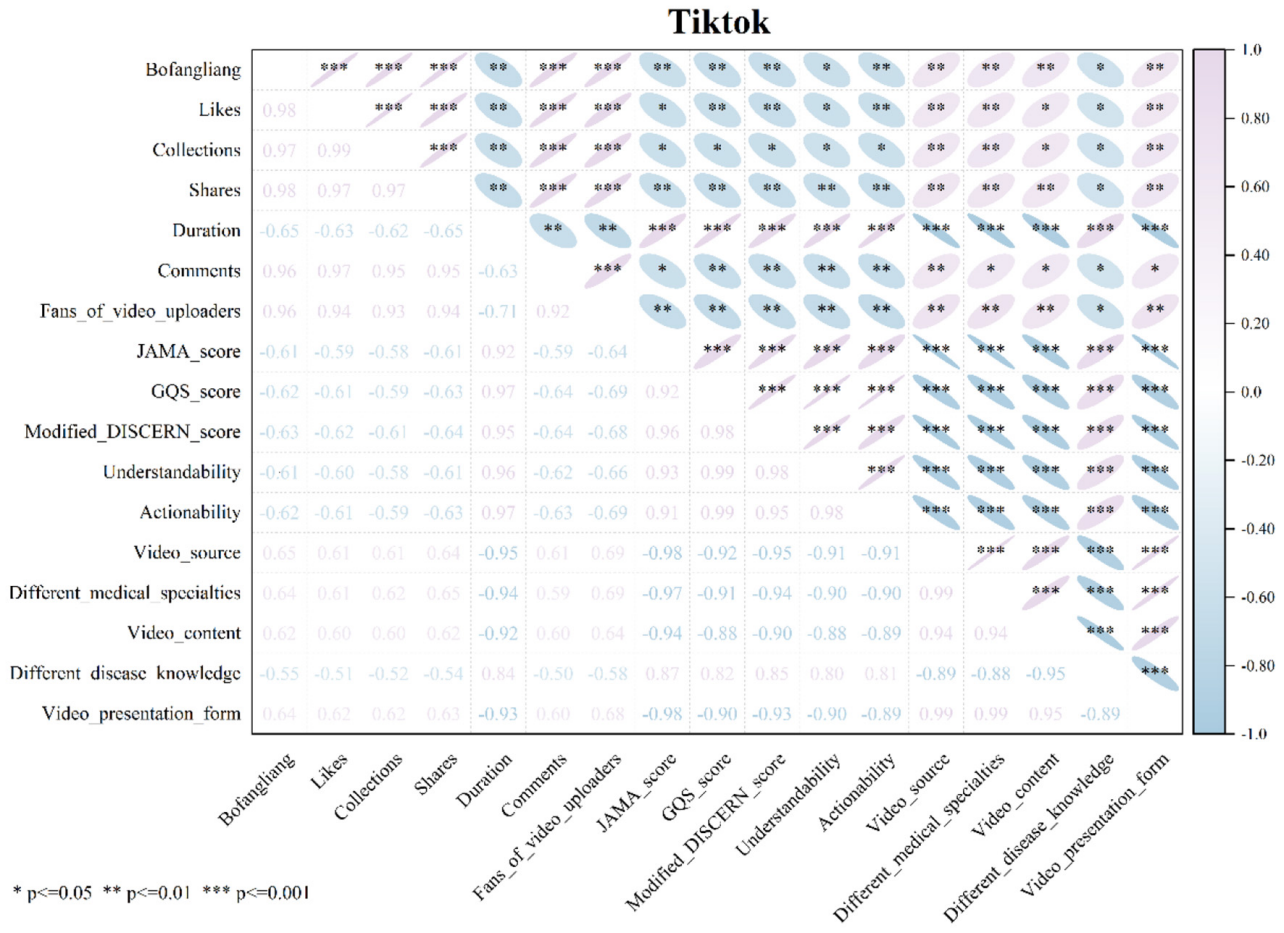
Spearman correlation analysis of variables in TikTok short videos.

As shown in Figure 5, Spearman correlation (ρ) analysis reveals the relationships among different video variables on TikTok. The results show that on TikTok, view counts, likes, comments, favorites, shares, and follower numbers are all significantly positively correlated with each other (ρ range: 0.92–0.96, all *P* < 0.001), in addition, a pronounced inverse association was observed between the number of followers and all video quality scores (JAMA score, GQS score, modified DISCERN score, PEMAT score), with Spearman's ρ values ranging from −0.64 to −0.69 and all *P* values < 0.01. Furthermore, it is noteworthy that video duration showed a significant positive correlation with all video quality scores (ρ range: 0.92–0.97, all *P* < 0.001), whereas video duration was significantly negatively correlated with all short video engagement metrics (views, likes, comments, favorites, and shares) (ρ range: −0.65 to −0.62, all *P* < 0.01). High-quality heat stroke-related short videos were associated with low traffic, and this phenomenon was most pronounced on TikTok when compared to the other two platforms (BiliBili and Kwai).

#### BiliBili: the balance between depth and popularity

**Figure 6:**
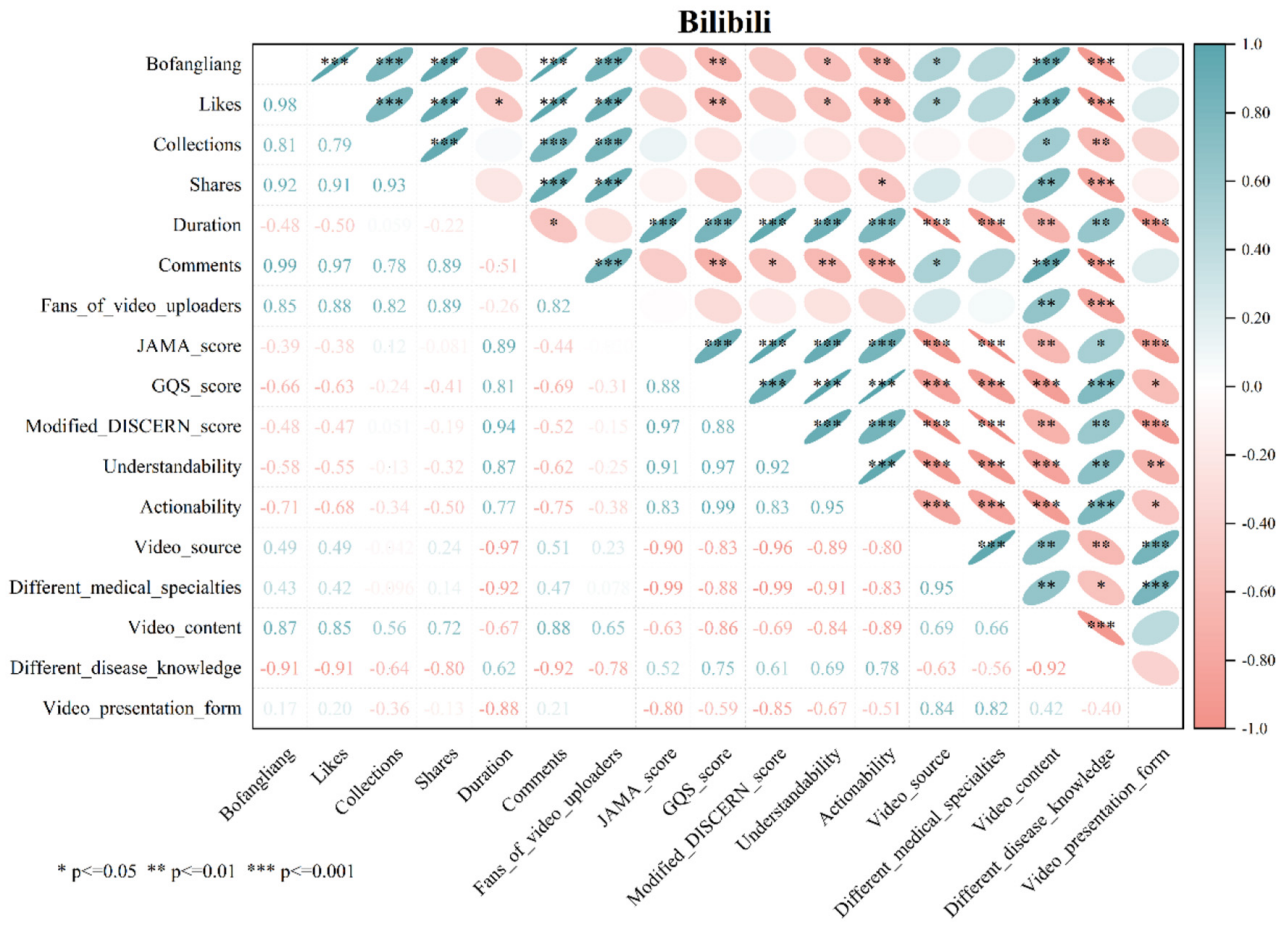
Spearman correlation analysis of variables in BiliBili short videos.

Like other platforms, BiliBili exhibits a distinct characteristic of very strong positive correlations both among its video interaction indicators (such as play count, likes, and favorites) and among its video quality measures (such as JAMA score and GQS score) (Figure 6). It is worth noting that video length was strongly positively associated with all video quality evaluation metrics (e.g., GQS score, Modified DISCERN score) (ρ: 0.77–0.94, all *P* < 0.001). Nevertheless, despite the association between longer duration and higher quality, a slight negative correlation was observed between interaction indicators (play count and likes) and quality ratings (GQS score and PEMAT score), with some relationships marked by asterisked significance. This implies that professionally produced, high-quality content often garners lower audience interaction compared to less professional material, although the magnitude of this negative association is substantially smaller than what was seen on TikTok.

#### Kwai: the significant independence between content quality and the traffic distribution mechanism

**Figure 7:**
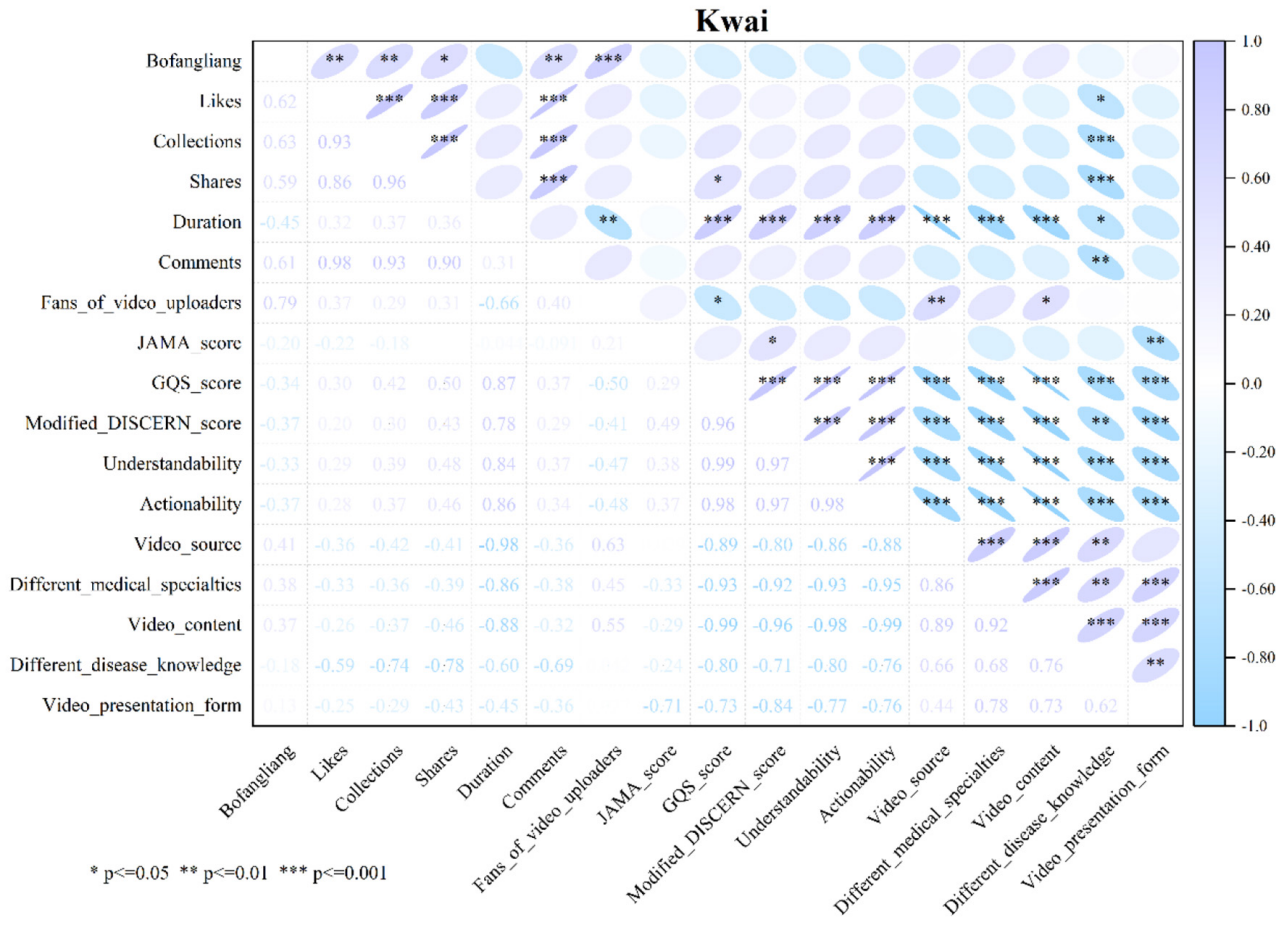
Spearman correlation analysis of variables in Kwai short videos.

In Kwai, the correlation heatmap shows that nearly all associations between video interaction indicators (such as likes, favorites, and shares) and quality ratings (e.g., JAMA score, GQS score, Modified DISCERN score) are extremely faint in color and devoid of significance indicators (asterisks). This suggests that there is no statistically meaningful relationship between the professional quality assessments of videos and their user popularity. Put differently, on Kwai, high-quality medical science videos do not benefit from algorithmic promotion nor are they disadvantaged in terms of traffic allocation, reflecting a pronounced disconnection between the two evaluative frameworks. While video length continues to show a positive association with quality ratings (ρ: 0.78–0.87, all *P* < 0.001), it does not exhibit a statistically significant relationship with most interaction indicators (such as likes, shares, and comments). This further underscores the stochastic nature of traffic allocation on the platform, indicating that short-form and long-form videos have comparable capacities for gaining traffic (Figure 7).

## Discussion

### Principal findings

In this study, we analyzed the content, reliability, and quality of heatstroke-related short videos from the major sharing platforms TikTok, BiliBili, and Kwai. Among these, videos on the Kwai platform showed the highest user engagement, gaining more likes, comments, favorites, and shares. However, in terms of overall video quality, heatstroke-related short videos on TikTok and BiliBili outperformed those on Kwai, achieving relatively higher JAMA, GQS, modified DISCERN, and PEMAT scores. Nevertheless, the overall quality of heatstroke-related short videos remains unsatisfactory. Physicians and news agencies were the main uploaders, focusing primarily on disease knowledge; most videos concentrated on describing symptoms and treatment, and mainly used visual imagery as the presentation mode. Across the three major platforms, user engagement indicators (such as views, likes, shares, and favorites) and uploader follower counts exhibited varying degrees of the Matthew effect—content with high transmissibility is prone to forming a positive feedback loop through user interaction behaviors, thereby accelerating its diffusion (18). In addition, the raters' scores in this study showed good consistency across all items, and the internal consistency reliability was also high.

### Quality of heatstroke short videos

Although public attention to heat stroke prevention and treatment information is increasing, this study found that even though physicians and news organizations are the main content contributors, the overall medical quality of short videos remains unsatisfactory. Notably, the quality disparities among platforms reflect the profound influence of review mechanisms and algorithmic ecosystems. Although heatstroke-related videos on Kwai had higher view counts, likes, shares, and favorites than those on TikTok and BiliBili. The overall quality and reliability on Kwai did not improve in proportion to the higher interaction metrics. Compared with Kwai, TikTok, and BiliBili had significantly higher JAMA, GQS, and modified DISCERN scores. This may be because TikTok and BiliBili have stricter qualification certification processes than Kwai for physicians and news agencies, allowing only certified individuals or organizations to publish health-related content, with the possibility of revoking certification for violations at any time (9). Such an initiative plays an important role in enhancing the quality and trustworthiness of short-video content. Furthermore, results indicated that the share of physician contributors was much greater on TikTok than on BiliBili or Kwai, respectively. Source type emerged as a critical determinant of heatstroke video quality; physician-produced educational content was notably more authoritative and professional, with stronger scientific grounding (evidence-based content, logical flow), greater practicality (clear procedural guidance, risk warnings), and superior audience intelligibility. Additionally, differences in user profiles across platforms (e.g., the distribution of urban vs. rural users) may lead to strategic divergence on the content supply side: some platforms might lower their requirements for professional depth to cater to the preferences of lower-tier markets, thereby achieving broader dissemination. For instance, TikTok and BiliBili enjoy high popularity in metropolitan and economically developed regions, whereas Kwai's user base is concentrated in less developed locales, including third-tier cities and rural communities (19). The interplay between user demographics and platform content standards can create a self-reinforcing cycle—users in less developed areas are more likely to contribute lower-quality content, and as platforms profit from the traffic such content generates, their motivation for rigorous moderation and quality enhancement may diminish (20). Partial results of this study suggest that the algorithm's varying emphasis on “video length” could be a critical constraint on the quality of popular science materials. Video length is a common feature associated with the integrity of medical information, reflected in high-quality ratings. Nevertheless, the “quality–traffic synergy” pattern observed on BiliBili—where long videos coincide with high engagement—was not reproduced on the other platforms. Especially on TikTok, high-quality videos were subject to a notable traffic disadvantage (negative correlation) because of their extended length. Such mechanisms suggest that the prevalent low quality of heat stroke-related popular science videos is not solely attributable to creators' abilities, but largely driven by platform algorithms' inherent bias toward “fragmented” and “high-arousal” content.

Accordingly, platforms might enhance the overall standard and trustworthiness of their short videos by pursuing three key strategies. First, collaborate with accredited medical associations to implement rigorous credentialing for heatstroke educational videos, accompanied by recognizable certification labels. Second, introduce a specialized “heatstroke” content hub on short-video platforms and enhance recommendation algorithms to surface credential-verified videos prominently in section-specific searches. Finally, integrate medical specialists or advanced AI systems into the video moderation workflow, enforcing strict action against misinformation and pseudoscience, thereby cultivating an authentic and science-driven educational environment.

### The significance of this study

As online popular science expands and public demand for health information rises, medical expertise is increasingly entering mainstream public awareness. Digital health education benefits patients by boosting their understanding of disease-specific knowledge and strengthening their awareness of health care practices. For instance, in a randomized controlled study, online video-based education for atopic dermatitis (AD) management markedly enhanced patient knowledge (questionnaire scores 3.05 vs. 1.85, *P* = 0.011) compared to written materials, and yielded better clinical outcomes (severity scale reduction of 3.30 vs. 1.03, *P* = 0.0043) (21). Furthermore, research has shown that online health education videos can likewise enhance patients' health consciousness. In a randomized controlled study on dietary adherence in diabetes, after 3 months of intervention, both face-to-face and video-based education groups achieved significant improvements in body weight, blood glucose, and blood lipid profiles compared with controls. Except for total cholesterol, no significant differences were observed between the two groups, and neither intervention significantly affected fasting glucose, LDL-C, or HDL-C levels. These findings suggest that video-based education is comparable in effectiveness to traditional face-to-face instruction (22). Consequently, online video-based education is becoming a significant and indispensable element in empowering patients to better manage their health. Nevertheless, the quality of videos warrants particular emphasis during this process. For instance, systematic assessments of cardiac rehabilitation-related videos indicate that even on global platforms like YouTube, low-quality material is widespread (23). In this context, disseminating precise and readily available health education videos on heat stroke could play a pivotal role in public health by enhancing early detection, prevention, and management capabilities for the disease.

### Strengths and limitations

This study systematically gathered heatstroke-related content from the three major short-video platforms—TikTok, BiliBili, and Kwai—providing a broad representation of the general landscape of heatstroke-related health information available online. Additionally, credentialed healthcare experts evaluated the quality, reliability, clarity, and actionability of the videos using JAMA, GQS, modified DISCERN, and PEMAT scoring systems. Furthermore, to boost analytical rigor, Spearman's rank correlation was applied to examine relationships among multiple video variables. The results can guide the public in selectively choosing high-quality, trustworthy heatstroke-related videos, enabling more effective acquisition of accurate health knowledge on the condition. Despite these strengths, there are notable limitations. First, as a cross-sectional study, evaluations were limited to a single time point; longitudinal research could provide deeper insights into how video content evolves over time. Second, the study focused solely on Chinese-language content, limiting generalizability; incorporating data from the international TikTok might enable valuable cross-cultural analyses. Third, assessments based on JAMA, GQS, and modified DISCERN metrics inevitably contain some subjective elements. Lastly, although data collection was conducted using newly created platform accounts, the possible biases arising from short video selection and inter-reviewer discrepancies should be given due consideration.

## Conclusion

In summary, the overall quality of heat stroke science popularization short videos on the three major platforms still needs improvement, with TikTok and BiliBili outperforming Kwai in terms of reliability. We propose that refining content quality evaluation systems and incentivizing professional involvement, combined with developing differentiated communication strategies based on each platform's algorithmic features (e.g., duration–traffic dynamics), could represent a viable pathway to boosting the efficacy of health information outreach. Future research should conduct longitudinal tracking to monitor changes in quality and develop AI-based automated assessment tools, thereby establishing a more objective and scalable evaluation system.

## Data Availability

The raw data supporting the conclusions of this article will be made available by the authors, without undue reservation.
