# Supplementary material for: Videos in short-video sharing platforms as sources of information on heatstroke: a cross-sectional content analysis study
Source: Front Public Health. 2026 Apr 9;14:1714828. doi: 10.3389/fpubh.2026.1714828 (PMC13104647; doi:10.3389/fpubh.2026.1714828)
Supplement: Supplementary file 1 [file Supplementary_file_1.docx]

**Table S1.** **Information classification of video content.**

| Video source |  |
| --- | --- |
| Physicians | Persons with formal qualifications in medical or healthcare (such as licensed physicians, etc.) or relevant academic background (professional medical researchers, etc.). |
| Independent users | Those who have not received formal medical or health care training |
| News agencies | A news organization that specifically collects and provides news articles, pictures and materials. |
| Others | Other sources of videos related to heat stroke include medical institutions and medical enterprises. |
| Different medical specialties |  |
| Western medicine practitioner | Clinical doctors in the field of Western medicine who specialize in treating heat stroke |
| TCM^a^ practitioner | Clinicians in traditional fields such as traditional Chinese medicine |
| Video content |  |
| Disease knowledge | The video mainly covers information related to heat stroke, including its epidemiology, pathogenesis, diagnosis, treatment and prevention. |
| Personal experience | Videoclipsofdocumentaryrecordsofillnessbyindividualsorfromothers |
| Others | Other videos related to heat stroke, such as advertisements and variety shows. |
| Different disease knowledge |  |
| Symptom | Increased body temperature, loss of consciousness, rapid breathing, accelerated heartbeat, etc. |
| Treatment | Transfer the environment, rapidly lower the temperature, correct the disorder of the internal environment, and provide support for organ functions, etc. |
| Prevention | Improving living environment, protecting outdoor work, ensuring reasonable and healthy diet and sleep, etc. |
| Pathogenesis | Temperature regulation imbalance, heat injury, inflammatory response and oxidative stress, etc. |
| Definition | Describe the concept of heat stroke, etc. |
| Others | Etiology, risk factors, etc. |
| Video presentation form |  |
| Expert monologue | The expert, from a first-person perspective, independently expounds on the viewpoints, knowledge, experiences, and research results related to heat stroke. |
| Visual pictures and literature | The dynamic images are presented in the form of static visual pictures. |
| Vlogs of patients | Record the patient's own perspective on the treatment process, experiences and so on. |
| Dialogue | Create and present the video content related to heat stroke through the form of conversations between two or more people |
| Animation | By leveraging animation production technology, the knowledge about heat stroke and other related contents are presented in a dynamic and fictional visual form and scenarios. |
| Others | Other presentation forms such as plot interpretation and teaching demonstrations |

**Table S2. The *Journal of American Medical Association* (JAMA) benchmark criteria.**

| Criteria (1 point for each) | Description |
| --- | --- |
| Authorship | Author and contributor credentials and their affiliations should be provided |
| Attribution | Clearly lists all copyright information and states references and sources for content |
| Currency | Initial date of posted content and subsequent updates to content should be provided |
| Disclosure | Conflicts of interest, funding, sponsorship, advertising, support, and video ownership should be fully disclosed |

**Table S3. Description of the Global Quality Score (GQS) scale.**

| Scale | Description |
| --- | --- |
| Poor quality (1 point) | Poor quality and poor flow of the site, most information missing, not at all useful for patients |
| Generally poor quality (2 point) | Generally poor quality and poor flow, some information listed but many important topics missing, of very limited use to patients |
| Moderate quality (3 point) | Moderate quality, sub-optimal flow, some important information is adequately discussed but others poorly discussed, somewhat useful for patients |
| Good quality (4 point) | Good quality and generally good flow, most of the relevant information is listed, but some topics not covered, useful for patients |
| Excellent quality (5 point) | Excellent quality and excellent flow, very useful for patients |

**Table S4. Description of the modified DISCERN score.**

| Criteria  (1 point for each) | Description |
| --- | --- |
| 1 | Is the video clear, concise, and understandable? |
| 2 | Are reliable sources of information used? (i.e., publication cited, speaker is specialist) |
| 3 | Is the information presented balanced and unbiased? |
| 4 | Are additional sources of information listed for patient reference? |
| 5 | Are areas of uncertainty/controversy mentioned? |

**Table S5. The Patient Education Materials Assessment Tool (PEMAT)**

**PEMAT- Understandability.**

|  | Item | Response Options | Rating |
| --- | --- | --- | --- |
| Topic: Content | | | |
| 1 | The material makes its purpose completely evident. | Disagree=0, Agree=1 |  |
| Topic: Word Choice & Style | | | |
| 3 | The material uses common, everyday language. | Disagree=0, Agree=1 |  |
| 4 | Medical terms are used only to familiarize audience with the terms. When used, medical terms are defined. | Disagree=0, Agree=1 |  |
| 5 | The material uses the active voice. | Disagree=0, Agree=1 |  |
| Topic: Organization | | | |
| 8 | The material breaks or "chunks" information into short sections. | Disagree=0, Agree=1,  Very short material=N/A |  |
| 9 | The material’s sections have informative headers. | Disagree=0, Agree=1,  Very short material=N/A |  |
| 10 | The material presents information in a logical sequence. | Disagree=0, Agree=1 |  |
| 11 | The material provides a summary. | Disagree=0, Agree=1,  Very short material=N/A |  |
| Topic: Layout & Design | | | |
| 12 | The material uses visual cues (e.g., arrows, boxes, bullets, bold, larger font, highlighting) to draw attention to key points. | Disagree=0, Agree=1, Video=N/A |  |
| 13 | Text on the screen is easy to read. | Disagree=0, Agree=1,  No text or all text is narrated=N/A |  |
| 14 | The material allows the user to hear the words clearly (e.g., not too fast, not garbled). | Disagree=0, Agree=1,  No narration=N/A |  |
| Topic: Use of Visual Aids | | | |
| 18 | The material uses illustrations and photographs that are clear and uncluttered. | Disagree=0, Agree=1,  No visual aids=N/A |  |
| 19 | The material uses simple tables with short and clear row and column headings. | Disagree=0, Agree=1,  No tables=N/A |  |

Total Points: _____________

Total Possible Points: _____________

Understandability Score (%): _____________

(Total Points / Total Possible Points x 100)

**PEMAT- Actionability**

|  | Item | Response Options | Rating |
| --- | --- | --- | --- |
| 20 | The material clearly identifies at least one action the user can take. | Disagree=0, Agree=1 |  |
| 21 | The material addresses the user directly when describing actions. | Disagree=0, Agree=1 |  |
| 22 | The material breaks down any action into manageable, explicit steps. | Disagree=0, Agree=1 |  |
| 25 | The material explains how to use the charts, graphs, tables, or diagrams to take actions. | Disagree=0, Agree=1,  No charts, graphs, tables, diagrams=N/A |  |

Total Points: _____________

Total Possible Points: _____________

Actionability Score (%): _____________

(Total Points / Total Possible Points x 100)
